# A case of bilateral pheochromocytoma during pregnancy

**DOI:** 10.1186/s12893-015-0041-1

**Published:** 2015-05-03

**Authors:** Kishu Kitayama, Shinichiro Kashiwagi, Ryosuke Amano, Satoru Noda, Go Ohira, Sadaaki Yamazoe, Kenjiro Kimura, Kae Hamamoto, Akihiro Hamuro, Masahiko Ohsawa, Naoyoshi Onoda, Kosei Hirakawa

**Affiliations:** Department of Surgical Oncology, Osaka City University Graduate School of Medicine, 1-4-3 Asahi-machi, Abeno-ku, Osaka Japan; Department of Metabolism and Molecular Medicine, Osaka City University Graduate School of Medicine, 1-4-3 Asahi-machi, Abeno-ku, Osaka Japan; Department of Obstetrics and Gynecology, Osaka City University Graduate School of Medicine, 1-4-3 Asahi-machi, Abeno-ku, Osaka Japan; Department of Diagnostic Pathology, Osaka City University Graduate School of Medicine, 1-4-3 Asahi-machi, Abeno-ku, Osaka Japan

**Keywords:** Pheochromocytoma, Pregnancy, Adrenal tumor, Bilateral, Adrenalectomy

## Abstract

**Background:**

Pheochromocytoma is a disease where catecholamines are secreted. If pheochromocytoma occurs during pregnancy, it can be difficult to diagnose because it is similar to pregnancy-induced hypertension. Furthermore, bilateral pheochromocytoma during pregnancy is even rarer than unilateral pheochromocytoma.

**Case presentation:**

A 32-year-old primigravida, who was 12 weeks’ pregnant, was aware of right abdominal discomfort. Masses in both adrenal glands were observed by abdominal ultrasonography. She was diagnosed with pheochromocytoma. Bilateral adrenalectomy was undertaken at 15 weeks’ gestation and she continued pregnancy. At 39 weeks’ gestation, a healthy male neonate was delivered. She was discharged on the 4th postpartum day.

**Conclusions:**

We present a case of bilateral pheochromocytoma during pregnancy that was diagnosed in the first trimester. Differentiating pheochromocytoma from pregnancy-induced hypertension is important. Early diagnosis and appropriate blood pressure management with medical treatment followed by surgical removal of the tumor results in good maternal and fetal outcomes.

## Background

Pheochromocytoma is an uncommon disease that exhibits a variety of sympathetic symptoms by secreting catecholamines [1,2]. Pheochromocytoma may also occur during pregnancy [3,4]. The symptoms of pheochromocytoma are similar to pregnancy-induced hypertension [5]. When pheochromocytoma occurs in pregnancy, maternal and fetal mortality is increased [6-8]. Therefore, early diagnosis and appropriate treatment are important because of these high risks. Moreover, bilateral pheochromocytoma during pregnancy is even rarer [9]. We report here an additional case of bilateral pheochromocytoma that was diagnosed in the first trimester.

## Case presentation

**Figure 1:**
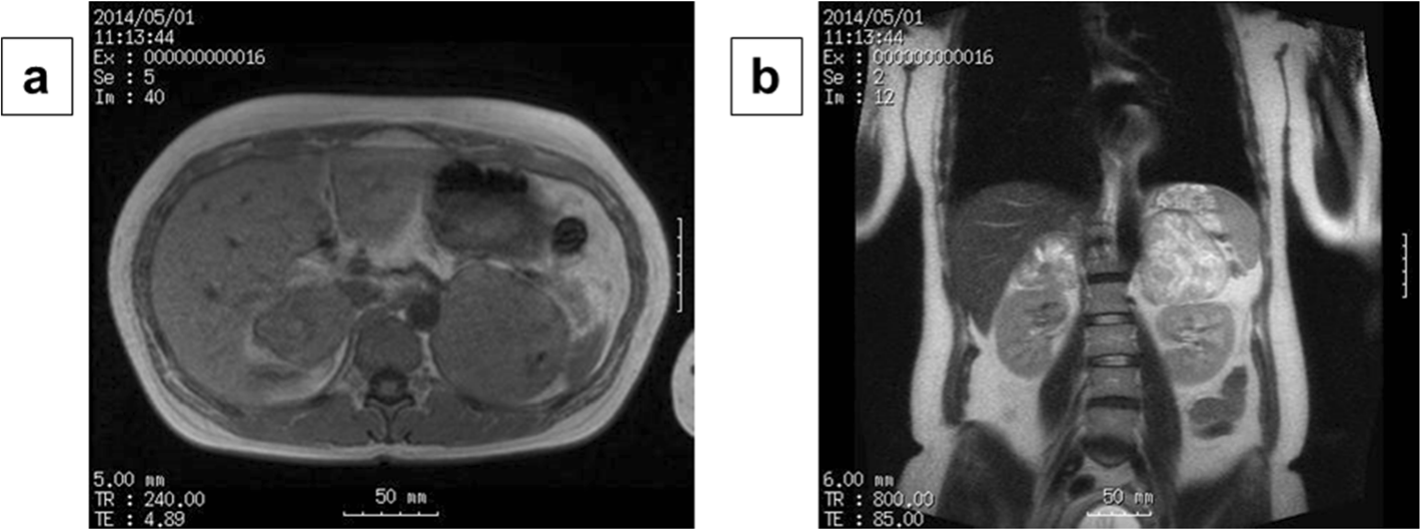
MRI findings: MRI shows bilateral adrenal masses with central necrosis. The right mass was 4.9 × 4.4 × 4.2 cm and the left mass was 7.3 × 6.1 × 7.5 cm, which were compatible with bilateral pheochromocytoma. A tumor exhibiting relative non-uniform low signals on T1 MRI was observed in both adrenal glands. Transverse plane **(a)**. Coronal plane **(b)**.

**Figure 2:**
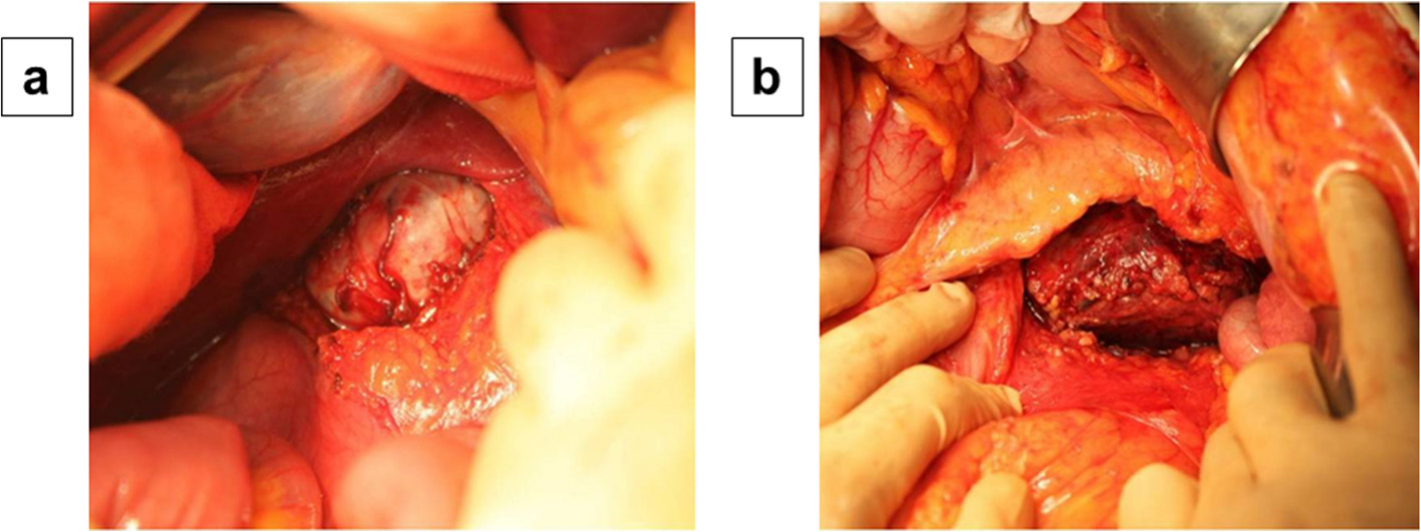
Surgical findings: Soft elastic tumor measuring 9 cm was palpable in the retroperitoneum at the inferior border of the pancreatic body. The tumor was removed en bloc without leaving any remnants **(a)**. After the left adrenalectomy, right adrenal adrenalectomy was then performed **(b)**.

**Figure 3:**
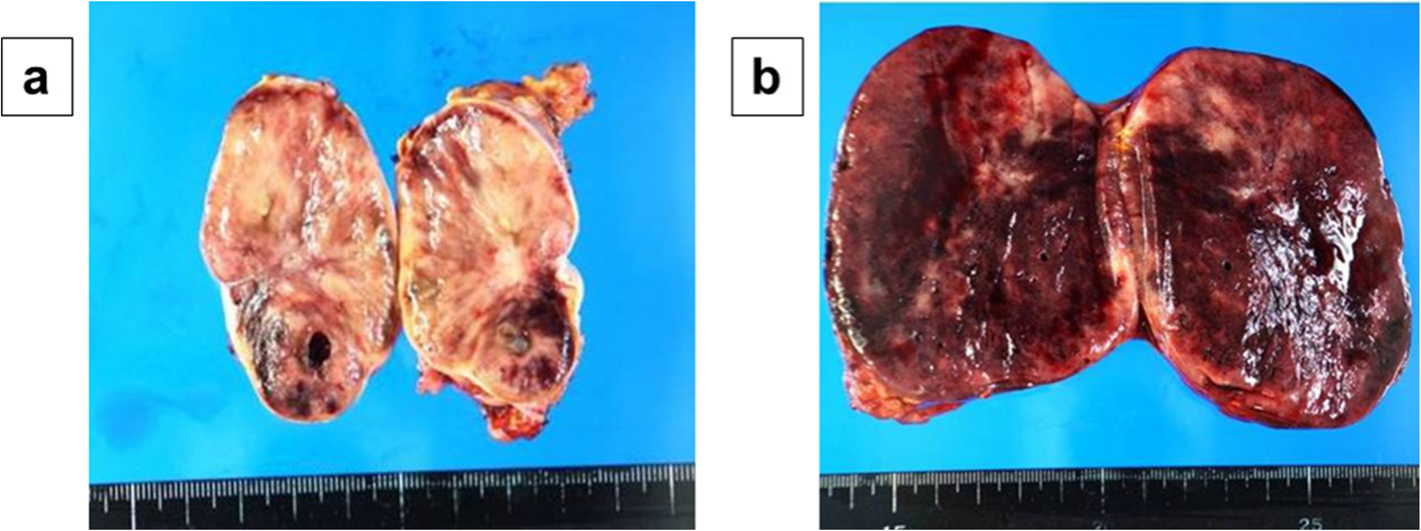
Resected specimen: The right tumor measured 5.5 × 4.5 × 3.5 cm, with a weight of 60 g **(a)**. The left tumor measured 9.0 × 8.5 × 5.5 cm, with a weight of 350 g **(b)**. The cut surface was a yellowish solid tumor, and areas of bleeding accompanied by necrosis in a branched pattern were observed.

**Figure 4:**
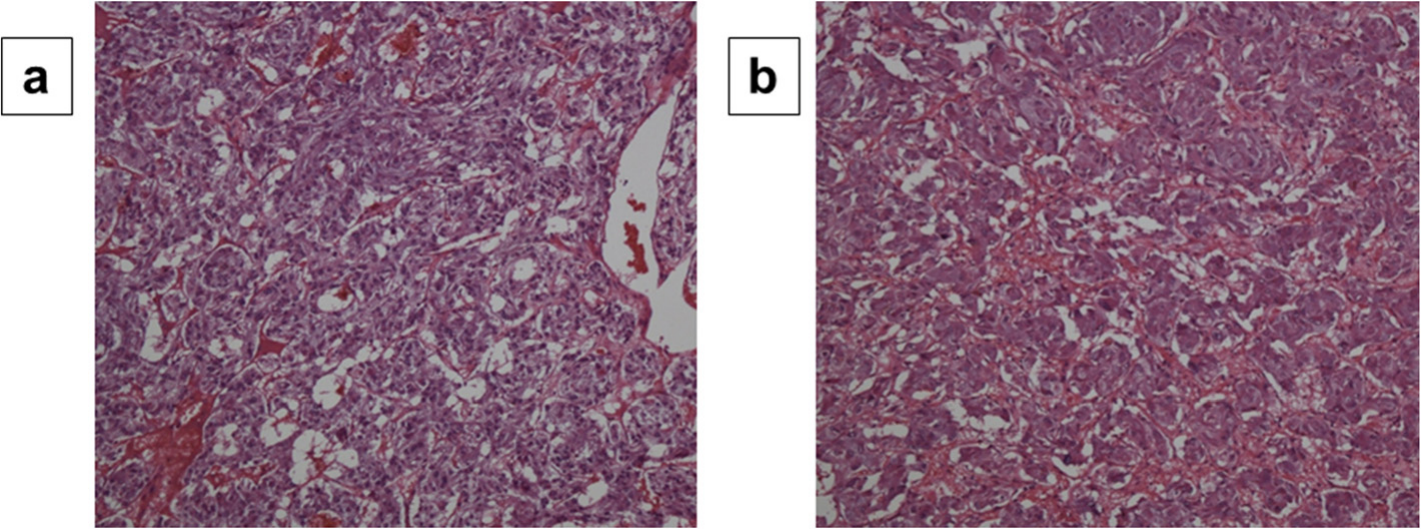
Histopathological findings. Histology of the masses confirmed pheochromocytoma of bilateral adrenal glands. Hematoxylin and eosin staining (x100), Right **(a)**, Left **(b)**.

A 32-year-old woman, primigravida, who was 12 weeks’ pregnant, initially consulted a practitioner with awareness of right abdominal discomfort. She was pointed out bilateral adrenal tumor by abdominal ultrasonography. She was diagnosed with pheochromocytoma by blood examination and consulted our hospital. There were no signs of preeclampsia. Her other past history were unremarkable. A physical examination showed a temperature of 35.7°C and a respiratory rate of 16 breaths/min. Her blood pressure was 129/90 mmHg and pulse rate was 86 beats/min. Her heart and breath sounds were normal. The size of her uterus was consistent with 12 weeks of gestation and fetal heart rate was 148 beats/min. There were no palpable masses in the thyroid, no uterine contractions, and no edema was detected. Major laboratory findings included a hematocrit of 38.8% and white blood cell count of 8,100/mm^3^, with 72.0% neutrophils and the platelet count was 391,000/mm^3^. The blood sugar level in the fasting was 87mg/dl. Serum free T3, free T4, and TSH levels were 3.07 pg/ml (normal, 2.30–4.00), 1.22 ng/dl (normal, 0.90–1.70), and 3.940 μunit/ml (normal, 0.500–5.000), respectively. Urinary albumin was negative. A 24-h urine sample demonstrated elevated metanephrine (14 mg/24 h; normal, 0.005–0.20 mg/24 h) and normetanephrine levels (12 mg/24 h; normal, 0.10–0.28 mg/24 h). An ultrasonogram showed a normal fetus, which was compatible with 12 weeks of gestation and solid masses. The crown-rump length was 59 mm and the biparietal diameter was 22 mm. Magnetic resonance imaging (MRI) confirmed bilateral adrenal masses with central necrosis (right, 4.9 × 4.4 × 4.2 cm; left, 7.3 × 6.1 × 7.5 cm), which were compatible with bilateral pheochromocytoma (Figure 1a, b). A tumor exhibiting relative non-uniform low signals on T1 MRI was observed in both adrenal glands. Alpha-adrenergic blockade with doxazosin mesylate 2 mg daily was initiated and blood pressure was maintained under 140/90 mmHg. Hydration was also started at 14 weeks of pregnancy. At 15 weeks’ gestation, exploratory laparotomy and bilateral adrenalectomy were performed. Noradrenaline was used by an anesthesiologist during an operation and maintained blood pressure. With regard to surgical findings, no hemorrhage or abnormal adhesions were observed in the abdominal cavity. A soft elastic tumor measuring 9 cm was palpable in the retroperitoneum at the inferior border of the pancreatic body (Figure 2a). The tumor was removed en bloc without leaving any remnants. After left adrenalectomy, right adrenal adrenalectomy was then performed (Figure 2b). The operative time was 167 minutes and the amount of bleeding was approximately 215 ml. The patient recovered uneventfully. The right tumor measured 5.5 × 4.5 × 3.5 cm, with a weight of 60 g (Figure 3a). The left tumor measured 9.0 × 8.5 × 5.5 cm, with a weight of 350 g (Figure 3b). The cut surface was a yellowish solid tumor, and areas of bleeding accompanied by necrosis in a branched pattern were observed. Histology of the masses confirmed pheochromocytoma of bilateral adrenal glands (Figure 4a, b). The postoperative course was uneventful but the patient also received daily prednisolone. Urinary metanephrine and normetanephrine levels were normalized on the 3rd day postoperation. Vital signs, the height of the uterus, fetal heart sounds, and urine protein levels were monitored accordingly. Postoperatively, the patient’s blood pressure was not controlled with any antihypertensive agents, with normalization of urinary catecholamine levels over time. The patient and her fetus were in good health. She did not show any signs of intrauterine growth retardation or pregnancy-induced hypertension. Therefore, she continued pregnancy as an outpatient. At 39 weeks and 1 day of gestation, a healthy male neonate weighing 2810 g was delivered by vaginal birth with Apgar scores of 8 and 9 at 1 and 5 min, respectively. She was discharged on the 4th postpartum day and was followed up. Her neonate was also discharged with a body weight of 2718 g on the 4th postpartum day. All date reported in the manuscript have been visualized and then approved by our University Hospital Ethics Committee and all procedures carried out on the patients were in compliance with the Helsinki Declaration. Moreover, the patient has given written explicit, express and unequivocal consent to publish her sensible date on our manuscript.

## Discussion

Pheochromocytoma is a disease that exhibits a variety of sympathetic symptoms by secreting catecholamines [1,2]. Most of the tumors in this condition are located in the adrenal medulla, but 10% are found in the sympathetic ganglia. Additionally, 10% of these tumors are extra-adrenal, 10% are bilateral, 10% are familial, and 10% are malignant. Therefore, pheochromocytoma is occasionally referred to as the “10% disease”. The incidence of bilateral pheochromocytoma is increased in familial cases. This incidence is increased to 80% in multiple endocrine neoplasia (MEN) IIA cases [10]. Pheochromocytoma during pregnancy is rare, with less than 300 reported cases [3,4,6-8,11]. Moreover, bilateral pheochromocytoma during pregnancy is even rarer than unilateral pheochromocytoma, with less than 20 cases reported [9]. Hypertension can be found in up to 98% of patients with pheochromocytoma [5]. However, the only symptoms and signs of catecholamine secretion in this case was awareness of right abdominal discomfort. Other sympathetic symptoms and signs were not found in our case, such as palpitations, tachycardia, sweating, seizure disorders, anxiety attacks, chest pain, dyspnea, nausea and vomiting, pallor, and flushing.

The diagnosis in our case was confirmed by measurement of 24-h urinary metanephrine and normetanephrine levels, as previously reported [12]. Measurement of 24-h urinary vanillylmandelic acid or catecholamines is also commonly used to confirm diagnosis of pheochromocytoma [3,5,10]. Urinary levels of catecholamines do not increase during normal pregnancy [3]. Localization of the tumor in this case was successful with magnetic resonance imaging (MRI) and abdominal ultrasonography, as in a previous report [10]. Computed tomography can be used to locate the tumor [3], but MRI has the advantage of lacking ionizing radiation [3,13]. Therefore, MRI is safe and used in pregnancy. However, iodine 131-metaiodobenzylguanidine scans is often necessary for localization [5]. Iodine 131-metaiodobenzylguanidine is used during the postpartum period [3]. These tests were not used in our case because MRI did not demonstrate extra-adrenal tumors, and postoperatively, the follow-up 24-h urinary metanephrine and normetanephrine levels decreased over time. Alpha-blockade was used as a first line medical treatment in our case, as described previously [3,5,10]. Maternal mortality from pheochromocytoma in pregnancy is high (4–17% or higher) if it is undiagnosed [3,7]. The diagnosis in our case was made in the first trimester, and therefore, maternal mortality did not occur. In a recent series, maternal mortality fell from 4–17% to 0–2% when the diagnosis was made antepartum [3,14].

The main treatment of pheochromocytoma is surgical removal [3]. The timing of the surgery is controversial and requires consideration on an individual basis. Surgery is less preferred during the first trimester because of the higher incidence of miscarriage. Adrenalectomy is recommended for second trimester cases. In the third trimester, surgery is delayed or often performed during cesarean section. In our case, pheochromocytoma was detected in the first trimester, and we continued management of blood pressure. Surgery was performed during pregnancy at 15 weeks’ gestation after waiting for development of the fetus. We waited until this time because enlargement of a gravid uterus makes operating technically difficult during an advanced pregnancy, and delaying the operation may be dangerous for both the mother and the fetus [3,5].

The majority of pheochromocytoma occurs sporadically. As mentioned above, approximately 10% of pheochromocytoma is familial, and it is usually bilateral. When bilateral pheochromocytoma is found, the associated syndromes should be searched for. These syndromes include MEN IIA (Sipple’s syndrome), MEN IIB (mucosal neuroma syndrome), neurofibromatosis, and von Hippel–Lindau disease [15]. However, dysmorphic features, including central obesity and skin striatum, were recognized. Furthermore, laboratory findings did not show these syndromes in our case.

## Conclusions

We present a case of bilateral pheochromocytoma during pregnancy that was diagnosed in the first trimester. Differentiating pheochromocytoma from pregnancy-induced hypertension is important. Early diagnosis and appropriate blood pressure management with medical treatment followed by surgical removal of the tumor results in good maternal and fetal outcomes.

### Consent

Written informed consent was obtained from the patient for publication of this case report including associated images.

## Abbreviations

T3: Triiodothyronine
T4: Thyroxine
TSH: Thyroid-stimulating hormone
VMA: Vanillylmandelic acid
MRI: Magnetic resonance imaging
